# Single-Cell RNA Sequencing of Human Pluripotent Stem Cell-Derived Macrophages for Quality Control of The Cell Therapy Product

**DOI:** 10.3389/fgene.2021.658862

**Published:** 2022-01-31

**Authors:** Hye-Yeong Jo, Hyang-Hee Seo, Dayeon Gil, YoungChan Park, Hyeong-Jun Han, Hyo-Won Han, Rajesh K. Thimmulappa, Sang Cheol Kim, Jung-Hyun Kim

**Affiliations:** ^1^ Division of Intractable Diseases Research, Department of Chronic Diseases Convergence Research, Korea National Institute of Health, Cheongju, South Korea; ^2^ Korea National Stem Cell Bank, Cheongju, South Korea; ^3^ Division of Healthcare and AI, Center for Precision Medicine, Korea National Institute of Health, Korea Centers for Disease Control and Prevention, Cheongju, South Korea; ^4^ Oneomics, Bucheon-si, South Korea; ^5^ Department of Biochemistry, Center of Excellence in Molecular Biology and Regenerative Medicine, JSS Medical College, JSS Academy of Higher Education & Research, Mysuru, India

**Keywords:** single-cell RNA sequencing, Pluripotent stem cell, macrophage, quality control, cell therapy product

## Abstract

Macrophages exhibit high plasticity to achieve their roles in maintaining tissue homeostasis, innate immunity, tissue repair and regeneration. Therefore, macrophages are being evaluated for cell-based therapeutics against inflammatory disorders and cancer. To overcome the limitation related to expansion of primary macrophages and cell numbers, human pluripotent stem cell (hPSC)-derived macrophages are considered as an alternative source of primary macrophages for clinical application. However, the quality of hPSC-derived macrophages with respect to the biological homogeneity remains still unclear. We previously reported a technique to produce hPSC-derived macrophages referred to as iMACs, which is amenable for scale-up. In this study, we have evaluated the biological homogeneity of the iMACs using a transcriptome dataset of 6,230 iMACs obtained by single-cell RNA sequencing. The dataset provides a valuable genomic profile for understanding the molecular characteristics of hPSC-derived macrophage cells and provide a measurement of transcriptomic homogeneity. Our study highlights the usefulness of single cell RNA-seq data in quality control of the cell-based therapy products.

## Introduction

Macrophages, a prominent cellular component of the innate immune system, are present throughout the body and play a pivotal role in tissue-specific homeostatic functions. They phagocytize and digest apoptotic cells, cellular debris, and microbes and produce soluble factors that modulate inflammatory-immune responses (either proinflammatory or anti-inflammatory responses) to govern host defense, tissue remodeling, healing and regeneration (Mosser and Edwards, 2008). The diverse functions of macrophages are attributed to their high plasticity properties governed by surrounding cues such as microbial products, dead cells and soluble factors (cytokines such as IFN gamma, IL-4, and IL-13) (Wynn et al., 2013). These unique functional features (antimicrobial activity, anti-inflammatory and tissue repair) render macrophages a promising candidate in a wide spectrum of cell-based therapeutic applications (Lee et al., 2016).

Macrophage-based cell therapy has been evaluated in patients and animal models to treat various inflammatory diseases, such as liver fibrosis (Starkey Lewis et al., 2019), multiorgan failure (Sharkey et al., 2019), cardiomyopathy (Henry et al., 2014), limb ischemia (Powell et al., 2012) and wound healing (Hu et al., 2017). Macrophages based immunotherapy is now being evaluated for treatment of solid malignant diseases. Macrophages, associated with tumors known as tumor associated macrophages (TAM), exerts pro-tumorigenic signals and promote invasiveness, angiogenesis, metastasis and therapeutic resistance (Zhou et al., 2020). To counter the TAM activity, adoptive transfer of modified or engineered macrophages is being evaluated to boost the endogenous immune response against tumors. Preclinical studies have successfully demonstrated the anti-tumorigenic efficacy of interferon gamma (IFNγ) activated macrophages or engineered macrophages to deliver Interferon alpha. In clinic, the therapeutic efficacy of IFNγ activated macrophages have been evaluated. Although the therapeutic success was limited, there were no serious side effects of the therapy, and this has opened avenues for the development of genetically engineered macrophage for cancer immunotherapy (Fidler, 1974; Alvey and Discher, 2017; Alvey et al., 2017). So far, most studies have explored the use of blood monocyte-derived macrophages, bone marrow-derived macrophages or CD34^+^ hematopoietic stem cell-derived macrophages for therapeutic applications (Fidler, 1974; Happle et al., 2014). However, there are several potential limitations for autologous macrophage-based cell therapies such as difficulties in obtaining sufficient cell numbers for adoptive transfer; effects of age and or pre-existing systemic condition on the differentiation potential and intrinsic biological activity of cells. To broaden the applicability of the macrophage based cell therapy, manufacturing macrophages would be beneficial for clinical applications and, the pluripotent stem cells (PSC) are one of the promising cell sources.

PSC such as human embryonic stem cells (hESC) and human induced pluripotent stem cell (hiPSC) undergo self-renewal and differentiate into any cell type (Thomson et al., 1998; Keller, 2005). This differentiation potential serves as a potent tool for disease modeling, drug screening, and toxicological assessment and provides novel insights into treatment alternatives for patients requiring cellular replacement therapies since they recapitulate human primary cells. Recently, we and others reported a method for generating and large-scale production of iPSC-derived macrophage-like cells in serum-free, feeder-free conditions (Han et al., 2019). iPSC-derived macrophages were phenotypically and functionally similar to the primary macrophages. Lately, a number of preclinical studies have successfully demonstrated the use of iPSC-derived macrophage for immunotherapy against various intractable diseases such cystic fibrosis (Mucci et al., 2018), cancers (Zhang et al., 2020) and respiratory bacterial infection (Ackermann et al., 2018). In addition, chimeric antigen receptor engineered macrophages derived from hPSCs showed promising effect against a solid cancer (Zhang et al., 2020). The preclinical success has greatly increased the enthusiasm for using engineered iPSC-derived macrophages in clinical setting specially for treatment of respiratory infections and cancer. Because macrophages are sensitive to surrounding micro-environments has high plasticity, determining the biological homogeneity of hPSC derived macrophages is essential for targeted genetic manipulation, therapeutic decisions, and successful clinical application.

Single-cell analysis has emerged as sensitive tool to reveal the biological heterogeneity and identifying the cell type-differences in a mixed population of cells. In this study, to decode the transcriptional information of hPSC-derived macrophage cells and uncover the cellular homogeneity, we performed unbiased large-scale in-depth characterization of hPSC derived macrophages by single-cell RNAseq analysis using 10× genomics platform. After filtering low-quality data with thresholds of mitochondrial gene expression (>20%), we analyzed the transcriptomic data of 6,230 cells. The filtered cells were highly homogenous, as judged by that fact that >90% of cells expressed the B2M gene (a typical housekeeping gene of hematopoietic cells) (Matsuzaki et al., 2015) and about 90% of cells expressed CD68 (a marker of classical macrophages) (Kunisch et al., 2004). Combined with FACs analysis, the single-cell analytical approach offers a superior means of ensuring cellular purity.

## Methods

### Cell Culture and Macrophage Differentiation

The PSC were differentiated into macrophages according to protocol as described previously by our group (Han et al., 2019). hESC H9 (WiCell) was maintained on a Matrigel-coated 35-mm dish using mTeSR 1 (STEMCELL), and the medium was replenished every day. ThehiPSC line CMC-hiPSC-003 was obtained from Korea National Stem Cell Bank (Kim et al., 2021) and maintained on a vitronectin-coated 35 mm dish using E8 media (STEMCELL). When colonies of both H9 or CMC-hiPSC-003 grew to approximately 500 μm in diameter, cellular differentiation was induced using mesoderm differentiation medium (APEL 2 supplemented with 1X insulin-transferrin-selenium-X [Invitrogen] and 100 ng/ml BMP4). After 2 days, 100 ng/ml BMP4 was replaced with 20 ng/ml BMP4 and incubated for 2 days. On day 4, BMP4 was replaced with 40 ng/ml VEGF and 50 ng/ml SCF. Two days later, the medium was replaced with hematopoietic differentiation medium (APEL 2 supplemented with 1X insulin-transferrin-selenium-X [Invitrogen], 50 ng/ml SCF, 10 ng/ml TPO, 50 ng/ml IL-3, 50 ng/ml IL-6, and 50 ng/ml Flt-3L). On day 15, floating cells were harvested and incubated in macrophage differentiation medium (RPMI 1640 supplemented with 100 ng/ml M-SCF).

### Cytospin Preparation and Diff-Quik Staining

Around 5,000 cells were centrifuged onto glass sides at 1,300 rpm for 10 min using a CYTOSPIN 4 system (Thermo Scientific). Slides were dried overnight and stained in Diff Quik (SYSMEX) in accordance with the manufacturer’s instructions.

### Flow Cytometry

Macrophages were fixed with 4% formaldehyde and washed with PBS. Cells were immunostained for CD11b, CD45, and CD86 for 20 min on ice. After washing with PBS, cells were resuspended in PBS and analyzed using an LSRFortessa system (BD Bioscience) and FlowJo software. The following antibodies were used: anti-CD45 (304052, BioLegend), anti-CD11b (301334, BioLegend), anti-CD86 (564544, BD Bioscience), and anti-CD14 (563743, BD Bioscience).

### Single-Cell RNA Sequencing

Single-cell capturing and barcoding to generate single-cell Gel Beads-in Emulsion (GEMs) was performed using the 10× Genomics Chromium platform in accordance with the manufacturer’s protocol. In brief, cell suspensions were loaded onto 10× Genomics Single Cell 3′ Chips with the reverse transcription master mix with RT Primer (TSO) (PN-310354). Cells were separated into GEMs along with gel beads coated with oligonucleotides, facilitating mRNA capture inside the droplets by 30 bp oligo-dTs after cell lysis and thus assigning barcodes to index cells (16 bp) with transcripts (10 bp UMI). After reverse transcription (RT), the barcoded cDNAs were amplified. Thereafter, a library was generated using the Single Cell 3′ Reagent Kit (v2 chemistry). We used the Illumina HiSeq 4000 system in stand-alone mode to sequence the libraries to obtain paired-end sequencing reads of 26 bp (read1) X 98 bp (read2). For barcode processing, UMI counting, and demultiplexing, we used the official 10× Genomics pipeline Cell Ranger v2.1.1. The raw base call files generated via the Illumina sequencers were demultiplexed into reads in FASTQ format using bcl2fastq v.2.20 (GEO GSE133935). The raw reads were then trimmed from the 3′ end, and the recommended number of cycles was determined for read pairs (Read1: 26 bp; Read2: 98 bp). The reads of each library were processed separately using the “cellranger count” pipeline to generate a gene-barcode matrix for each library. Therein, the reads were aligned to a human reference genome (version: hg19, GRCh37.p13).

### Statistical Analysis

The concatenated gene matrix, barcode matrix, and count matrix were imported into Seurat (Macosko et al., 2015) v4.0 (http://satijalab.org/seurat/) for data processing. Seurat is an R package designed for quality control, analysis, and evaluation of single-cell RNAseq data. After creating the Seurat object from feature expression matrix of the sample, we filtered out genes whose expression was detected in fewer than 5 cells, cells with less than 200 unique gene counts (nFeature_RNA) and less than 25% mitochondrial genes. The resulting data were normalized using “LogNormalize” method that normalizes the read counts by dividing by the total counts for that cell and multiplied by the scale factor. Subsequently, we identified top variable 2,000 features that are outliers using “variance stabilizing transformation (vst)” method. To determine the dimensionality of the dataset, we performed dimensional reduction of the data through principal component analysis. The statistical method JackStraw (in Seurat) was used to determine the number of significant components. We used the Shared Nearest Neighbor (SNN) modularization-based clustering algorithm to identify cell clusters (the “FindClusters” function, resolution = 0.5). Lastly, the principal component analysis was used to visualize the clustered cells via the Uniform Manifold Approximation and Projection (UMAP) dimension reduction. We additionally performed an unsupervised clustering for cell population identification using single cell RNA-seq data (ILoReg, version 1.2.0) (Smolander et al., 2021) to detect minor subpopulation of cell after log transformation of count data in Seurat object. The number of clusters was determined by maximum silhouette value, which indicated the stability of each cluster. The ILoReg package includes the function named “CalcSilhInfo” and “SilhouetteCurve” that allows us to evaluate the sample-cluster relationship for a selected number of clusters (k). We explored sample versus cluster assignments over a wide range of k using this function. We found k = 5 (or 9) to arguably be the best one. To identify top-ranked gene markers in each cluster, we also used the “FindAllGeneMarkers” function to access both differentially expressed features across the samples and significant gene markers per cluster. The mean cluster expression values (under all cell samples of each cluster) were used to construct a binary classifier prediction for a given gene.

### Data Records

Data are available in the GEO database under accession number GSE133935. The files comprise raw FASTQ files and a tab-separated matrix of read counts for each cell passing quality control filtering. BAM files can be generated using the supplied repository to process the FASTQ files using Cell Ranger v2.1.1.

### Code Availability

All analysis r-codes, datasets and usage notes are available at https://github.com/kimsc77/iMACs, including statistical analysis pipelines used to process the sequence data (UMI expression matrix), as well as scripts for dataset loading, data filtering, normalization, scaling, clustering, differential expression, and visualization.

## Results

### Preparation of Human hPSC-Derived Macrophage Samples and Generation of the Single-Cell RNAseq Dataset

**FIGURE 1:**
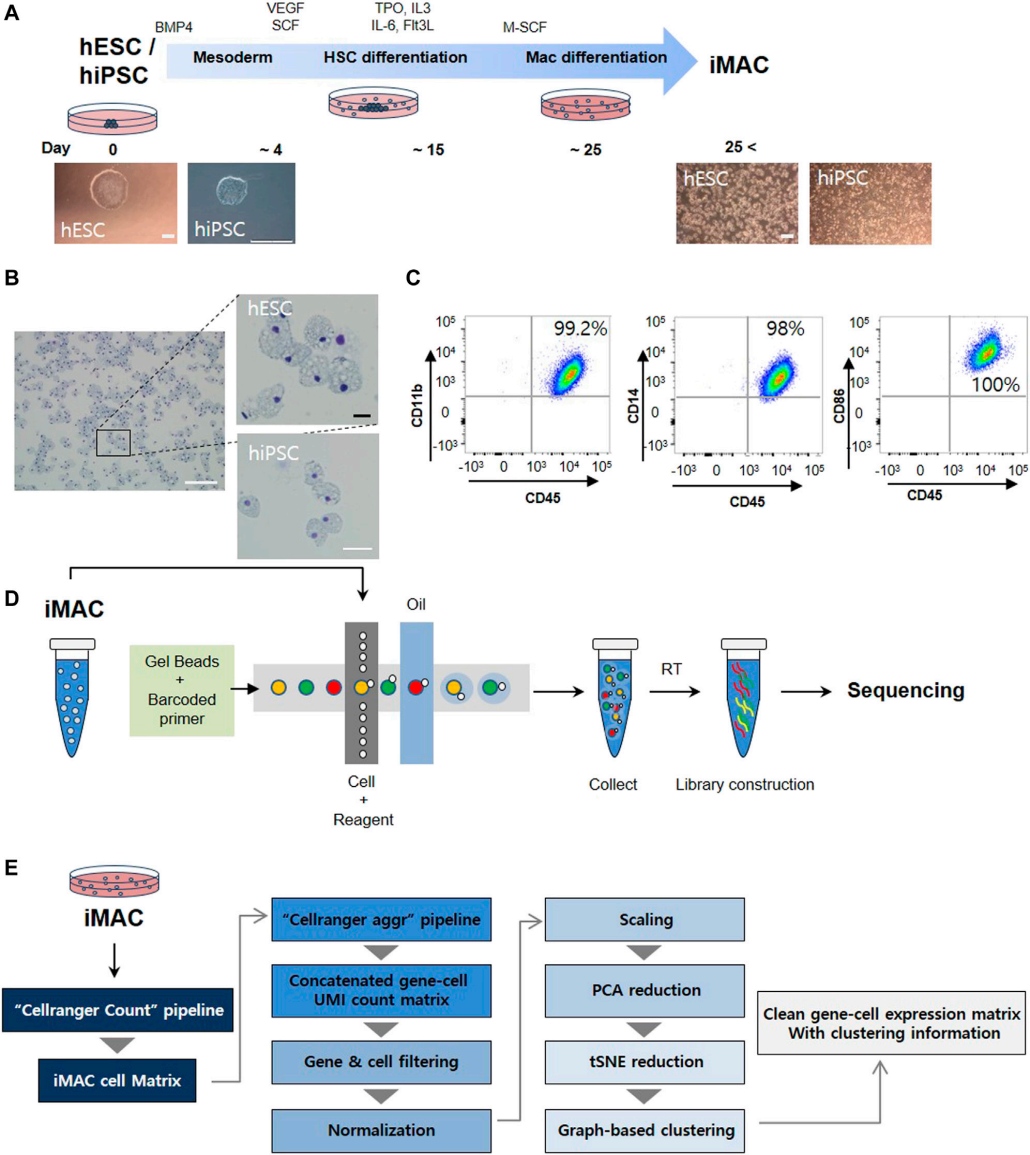
Overview of the study protocol. **(A)** A schematic representation of cell differentiation from human embryonic stem cells (hESC) or human induced pluripotent stem cell (hiPSC) to macrophages. **(B)** Typical morphologic features of macrophages from hESC and hiPSC are shown. **(C)** Macrophage markers (CD14, CD11b, CD86, and CD45) confirmed in hESC derived iMAC via flow cytometry. **(D)** Macrophage single-cell RNA sequencing libraries were generated using the Illumina HiSeq 4000 platform. **(E)** Pipeline for single-cell RNAseq analysis. Scale bar: white = 200 μm, black = 25 μm.

Human embryonic stem cell (hESC) and human induced pluripotent stem cell (hiPSC) were differentiated into macrophages (iMACs) following the method reported in our published study (Han et al., 2019) (Figure 1A). iMACs from the hESC and hiPSC had a comparable morphology with blood monocyte-derived macrophages (Figure 1B), and flow cytometry analysis revealed that more than 99% of iMACs were double-positive for the macrophage-specific markers CD11b, CD14, CD45, and CD86 (Figure 1C). After confirmation, we used the 10× Genomics Chromium platform to generate a single-cell RNA sequencing library of iMACs (Figure 1D). Thereafter, a gene-cell expression matrix was generated with clustering information (Figure 1E).

The distribution of gene counts (nGene), UMI counts and mitochondrial transcript levels is shown in Supplementary Figure S1A. To filter out the low-quality data potentially arising from damaged cells, we set tight thresholds for the data in accordance with mitochondrial gene expression (>20%) (Supplementary Figure S1B), and the high-quality data of 3,092 for hESC derived iMAC cells (hESC-iMAC) and 3,138 hiPSC derived iMAC cells (hiPSC-iMAC) were finally retained for analysis (Supplementary Table S1). To verify the characteristics of each cell, dimensional reduction of the data was conducted via principal component analysis. JackStraw analysis was performed, and PC 13 was selected for further analysis (Supplementary Figure S1C).

### Cell Homogeneity Evaluation Using Single-Cell RNAseq Analyzed by Seurat Method

**FIGURE 2:**
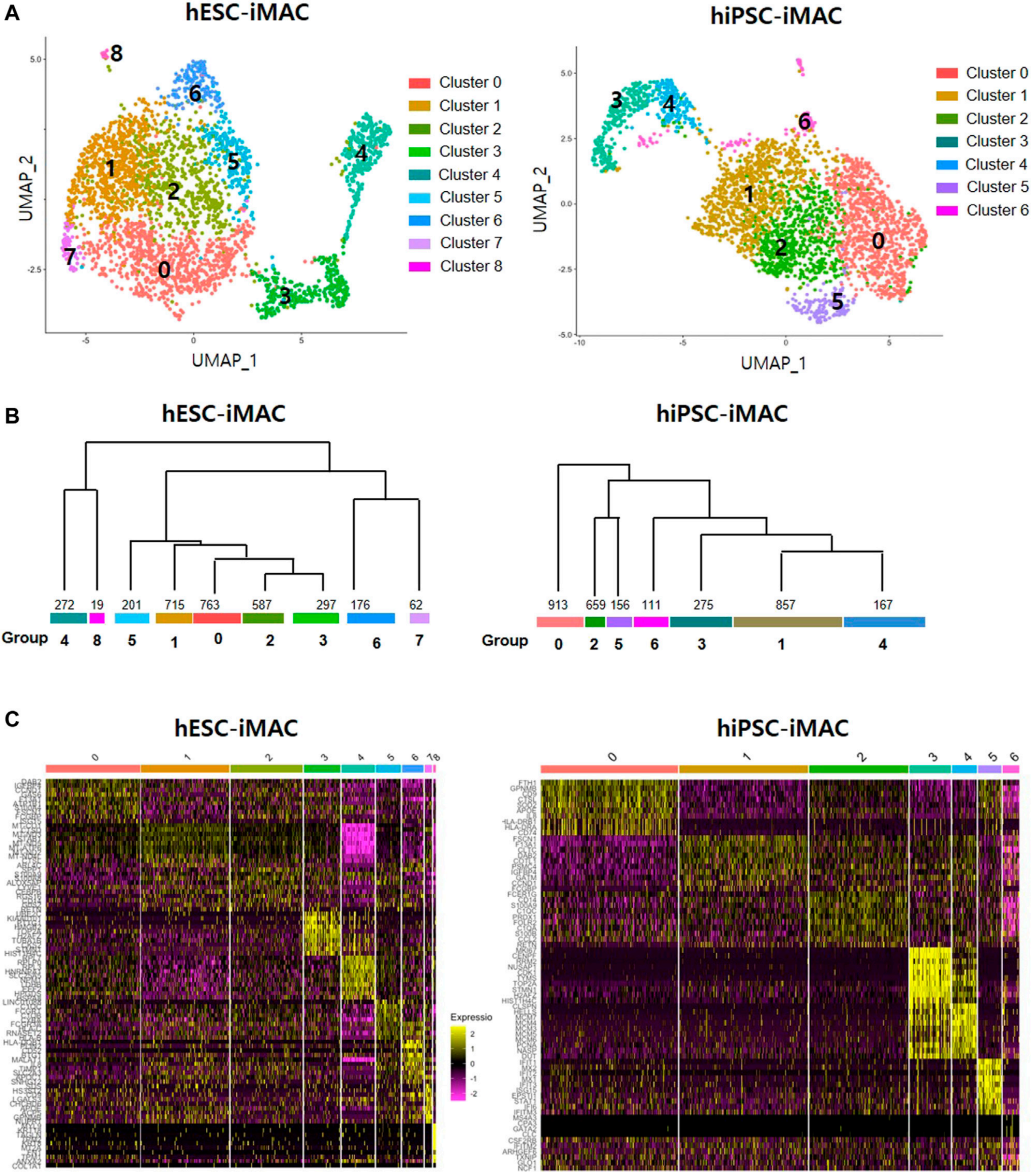
Cellular heterogeneity within the filtered induced macrophages (iMACs) analyzed by Seurat method. In total, 3,092 hESC derived iMACs (hESC-iMAC) and 3,138 hiPSC derived iMAC (hiPSC-iMAC) were analyzed *via* two-dimensional UMAP analysis to visualize their similarity. **(A)** The iMACs were grouped into 6 (hESC-iMAC) and 8 (hiPSC-iMAC) clusters. The color codes are as indicated. **(B)** The dendrogram showing associations among clusters. The bottom of the branch indicates the number of cells in each cluster. **(C)** Heatmap shows the expression of the top 10 genes of each group.

**FIGURE 3:**
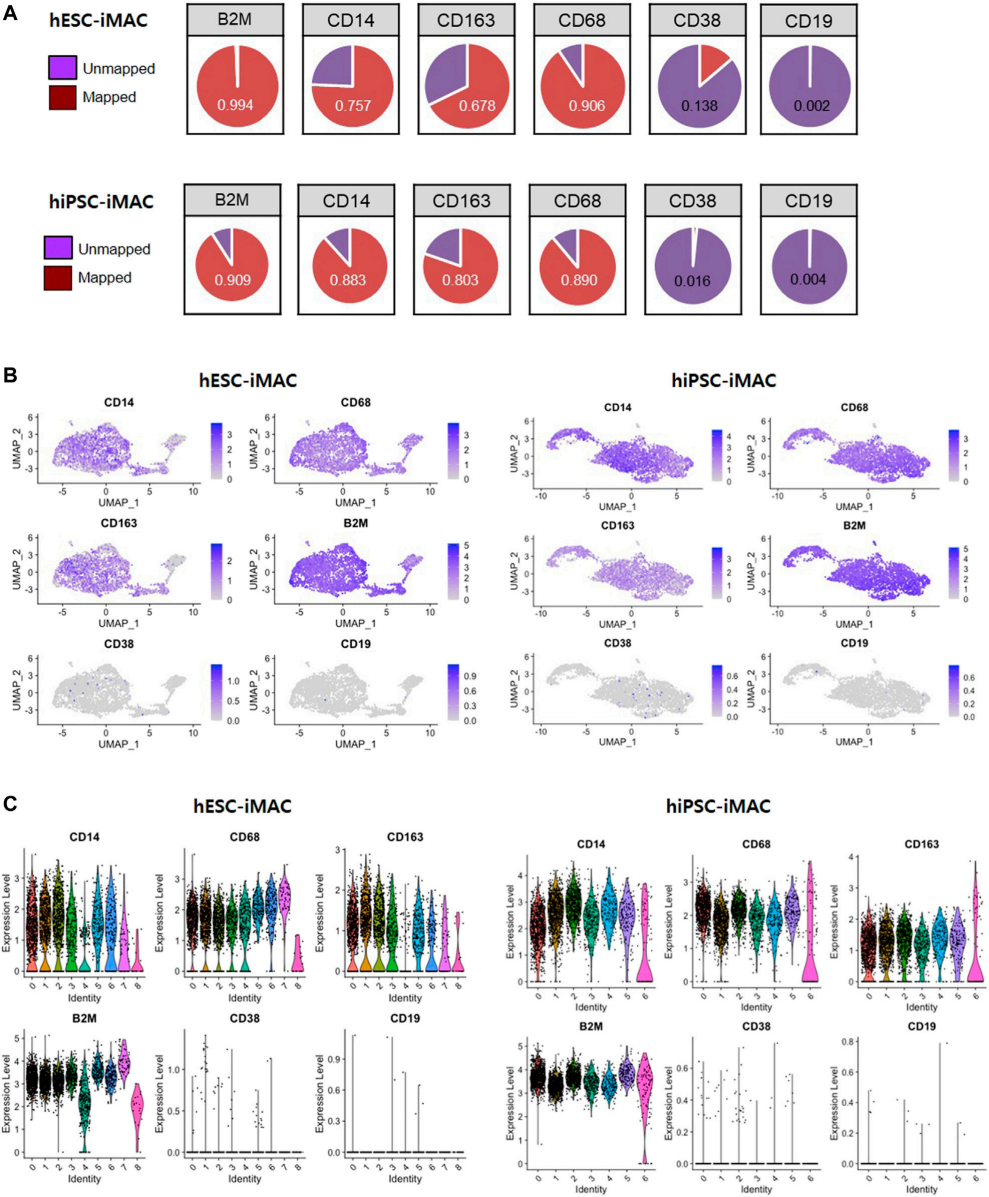
Expression level of known macrophage markers analyzed by Seurat method. **(A)** The proportion of cells expressing macrophage-positive and macrophage-negative marker genes and the number of mapped genes. Violin and jitter plots and UMAP plots for the expression of **(B)** macrophage-positive markers (CD14, CD68, CD163, and B2M) and **(C)** macrophage-negative markers (CD38 and CD19).

Clustering analysis of hESC-iMAC revealed nine distinct subpopulations comprising of 763, 715, 587, 297, 272, 201,176, 62, and 19 cells (Figures 2A,B). Clustering analysis of hiPSC-iMAC disclosed seven distinct groups comprising of 1,083, 747, 500, 323, 171, 121 and 121 cells (Figures 2A,B). Subsequently, we applied bottom-up agglomerative hierarchical clustering (Figure 2C). To assess the heterogeneity of the macrophage cell population based on transcriptome differences, we evaluated the distribution of macrophage specific markers and B or T cell markers within the clusters. Although macrophages contain specific markers based on their subtype and location, pan-macrophage markers such as CD45, CD14, CD86, CD68, CD163, CD11b, CD11c, CD80, CCR5, and B2M, are expressed by all macrophage populations. Therefore, we examined the expression of selected macrophage markers in individual hESC-iMAC and hiPSC-iMAC. We observed 99.3% of cells expressed B2M in hESC-iMAC and 90% of cells expressed B2M in hiPSC-iMAC; around 90.7% of cells were positive for CD68 in hESC-iMAC and 88% of cells were positive for CD68 in hiPSC-iMAC; 75.4% of hESC-iMAC and 80% of hiPSC-iMAC cells expressed CD14. In contrast, the expression of the lymphoid cell marker, CD38 and CD19 were rarely expressed at 14 and 0.2% for hESC-iMAC and 1.6 and 0.4% for hiPSC-iMAC, respectively (Figure 3A). Furthermore, we examined transcriptional dynamics in each cluster based on the expression of those markers. In the cluster of cells positive for macrophage markers (CD14, CD68, CD163, and B2M), the genes were upregulated in all clusters (Figures 3B,C). In contrast, the same set of genes were down regulated in all the cluster of cells expressing CD38 and CD19 (macrophage-negative markers) indicating high homogeneity of the cell population (Figures 3B,C).

### Identification of Major Subtypes in the iMACs Using Single-Cell RNAseq

**FIGURE 4:**
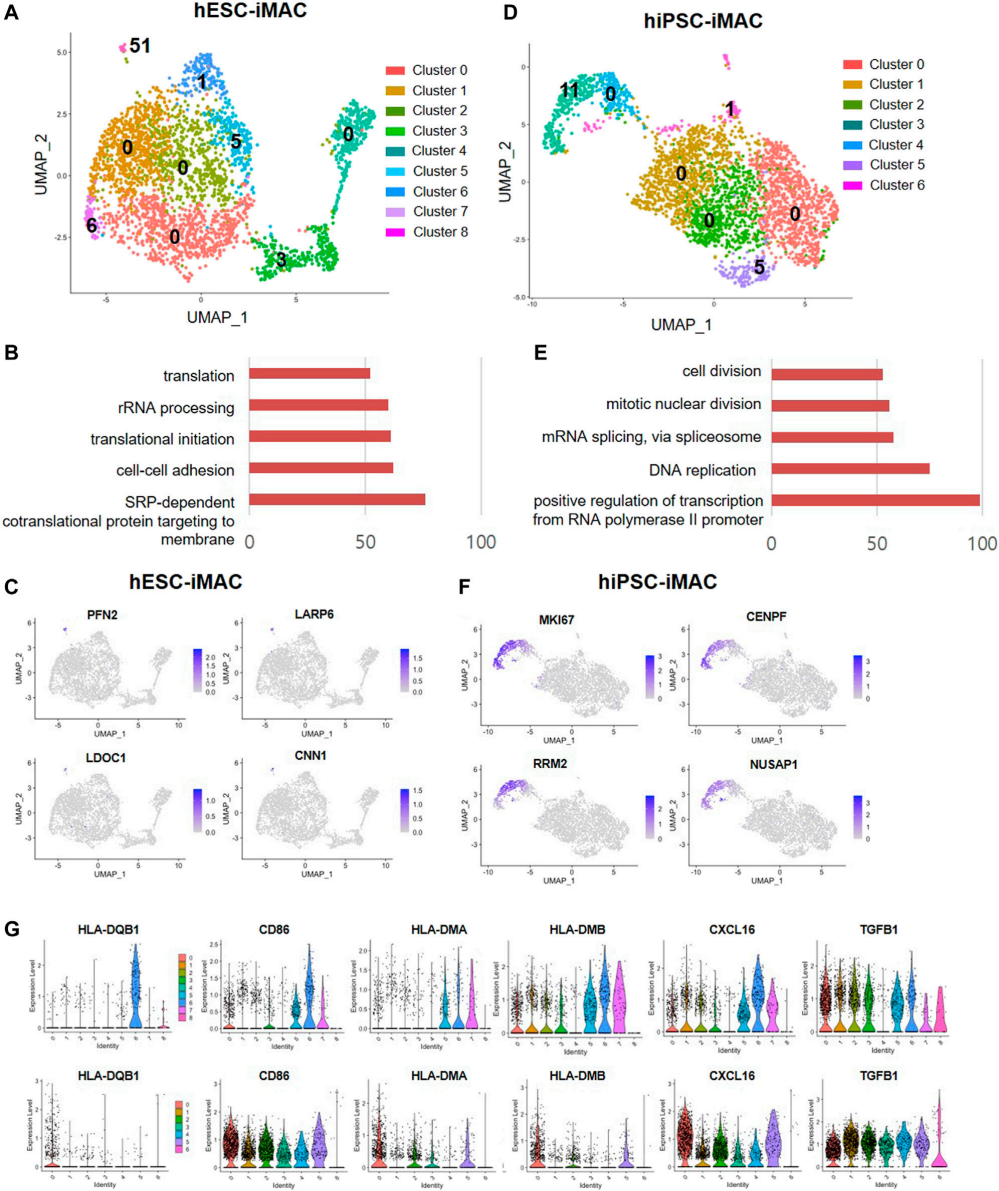
Differentially expressed genes and M1 and M2 macrophage marker genes in iMACs. **(A)** Number of DEGs of each cluster (Log2 fold change >2, *p* < 0.05, exclude mitochondrial genes) in hESC-iMAC. **(B)** Gene ontology (GO) analysis of DEGs in cluster 8 (hESC-iMAC) (GO FDR < 0.05, DEG count >10, Top 5) **(C)** UMAP plots for the expression of the top 4 genes in cluster 8 of hESC-iMAC. **(D)** Number of DEGs of each cluster (Log2 fold change >2, *p* < 0.05, exclude mitochondrial genes) in hiPSC-iMAC. **(E)** Gene ontology (GO) analysis of DEGs in cluster 3 (hiPSC-iMAC) (GO FDR <0.05, DEG count >10, Top 5). **(F)** UMAP plots for the expression of the top 4 genes in cluster 3 of hiPSC-iMAC. **(G)** Gene expression of M1 and M2 macrophage markers in each cluster.

Next, to identify subtype of cells in each cluster based on transcriptomic characteristics, we performed comparative analysis of differentially expressed genes (DEGs). Between the clusters 0, 1, 2, and 4, which accounts for 75.5% of the total cells [2,337 cells out of 3,092 cells (Log2 fold change> 2, *p* < 0.05)], we did not observe any significant difference in the DEGs. However, we found a significant difference in DEGs between the clusters 3, 5, 6, 7, and 8, which contained 24.5% of the total cells (Figure 4A; Supplementary Table S2). The cluster 8, which consisted of 1.6% of the total cells had more than 51 DEGs. To predict the features cluster 8, we performed GO analysis using DEGs of cluster 8. Overall, there was significant GO terms related to translation, cell-cell adhesion (GO FDR < 0.05, Top 5 ranking) (Figure 4B). The expression of selected DEG genes is displayed in UMAP plots (Figure 4C).

In consistent with hESC-iMAC, hiPSC-iMAC showed no significant DEGs in cluster 0, 1, 2, and 4 which accounts for 82.7% of total cells (2,596 cells out of 3,138 cells) (Figure 4D) indicating a homogenous transcriptomic feature. Unlike other clusters, cluster 3 showed 11 DEGs which related to cell division, mitotic nuclear division, RNA splicing, DNA replication and transcription (GO FDR<0.05, Top 5 ranking) in the hiPSC-iMAC data (Figure 4E; Supplementary Table S2). The expression of selected DEG genes of hiPSC-iMAC is displayed in UMAP plots (Figure 4F). Next, we evaluated whether clusters can be further classified into subpopulations based on known macrophage subtype markers of M1 and M2 (Figure 4G) of both hESC-iMAC and hiPSC-iMAC. MHC class II molecules (HLA-DQB1, HLA-DMA, HLA-DMB), CD86, and CXCL16 were used as M1 markers, and TGFB1 was used as M2 Marker. Violin plots showed that the expression of the MHC class II molecules HLA-DQB1 and CD86 was elevated in cluster 7 of hESC-iMAC. However, all the clusters in hiPSC-iMAC expressed CD86 and TFG beta to a similar level (Figure 4G). Therefore, there was no apparent distinction between M1 and M2 populations in the iMAC.

### Cell Homogeneity Confirmed by ILOReg Analysis Method

**FIGURE 5:**
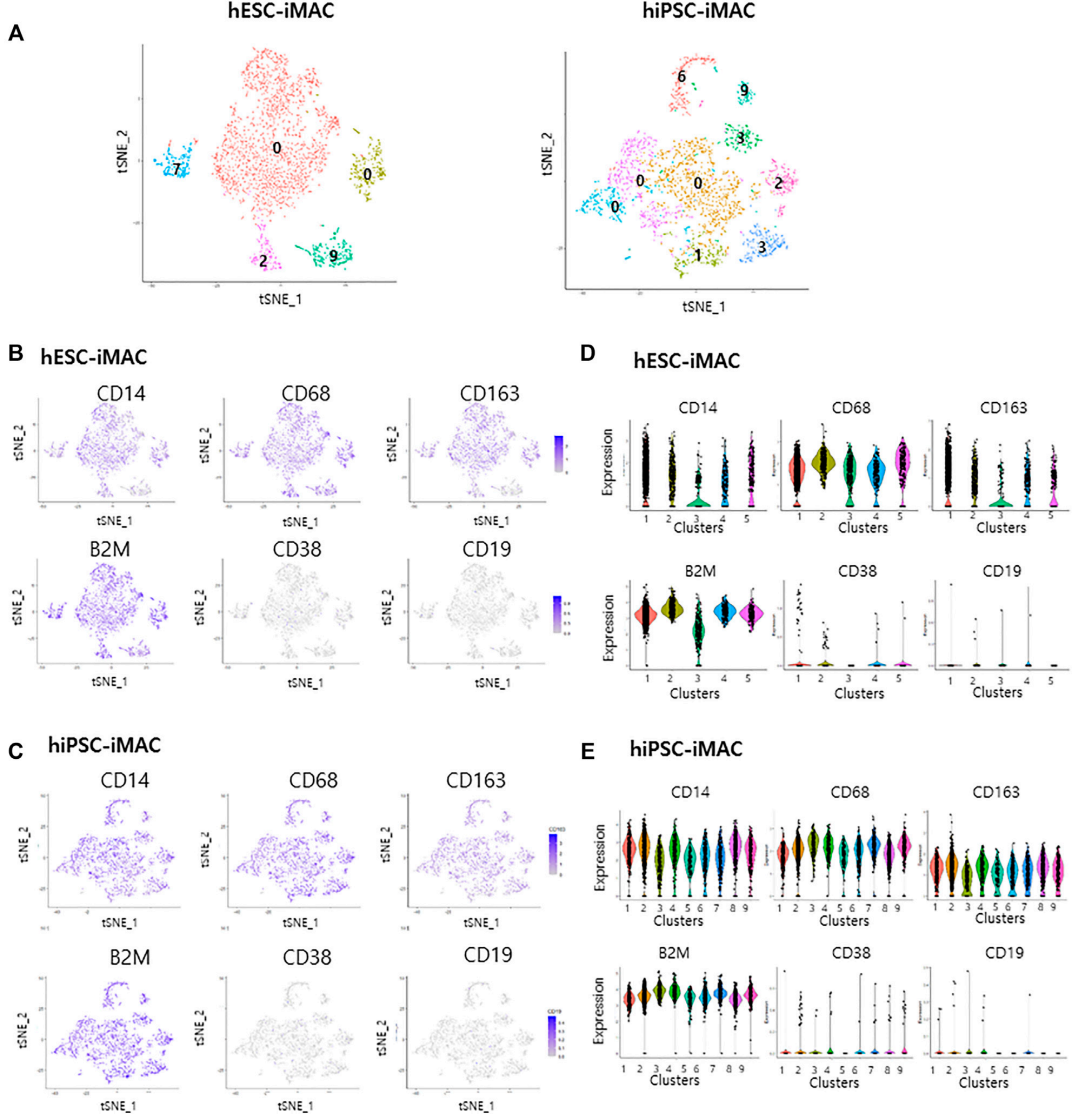
Cellular homogenity within the filtered induced macrophages (iMACs) analyzed by ILOReg methods. In total, 3,092 hESC derived iMACs (hESC-iMAC) and 3,138 hiPSC derived iMAC (hiPSC-iMAC) were analyzed *via* two-dimensional tSNE analysis to visualize their similarity. **(A)** Number of DEGs of each cluster (Log2 fold change >1, *p* < 0.05, exclude mitochondrial genes) in hESC-iMAC (left) and hiPSC-iMAC (right). **(B–E)** The proportion of cells expressing macrophage-positive and macrophage-negative marker genes and the number of mapped genes. **(B,C)** tSNE plots and **(D,E)** violin and jitter plots for the expression of macrophage-positive markers (CD14, CD68, CD163, and B2M) and macrophage-negative markers (CD38 and CD19).

A previous study pointed out how different statistical analytical methods impacts the DEGs output (Krzak et al., 2019). Therefore, to confirm the homogeneity of the cells, we additionally analyzed the data with another computational method ILoReg, that takes an alternative approach to dimensionality reduction by means of feature extraction (Smolander et al., 2021). Clustering of hESC-iMAC revealed 5 distinct subpopulations and, hiPSC-iMAC showed 9 distinct subpopulations (Figure 5A). There were no DEGs in any subgroups Log2FC > 2, *p* < 0.05 and small number of DEGs Log2FC > 1, *p* < 0.05 (Figure 5A; Supplementary Table S3), indicating that the individual cells have homogeneous transcriptomic feature. In addition, when we examined the transcriptional dynamics in each cluster based on the expression of macrophage markers, most of the clusters showed macrophage-positive markers (CD14, CD68, CD163, and B2M) (Figures 5B–E), and were negative for CD38 and CD19 markers (Figures 5B–E).

## Discussion

Cell-based therapies are a rapidly growing area of regenerative medicine. Macrophages are attractive candidate for cell-based therapeutics due to their diverse functionality (Andreesen et al., 1998; Lin et al., 2019; Perciani and MacParland, 2019) and currently, manufactured macrophages are being evaluated in clinic against various disorders (e.g., cancer and renal transplantation). The phenotypic and functional purity of the macrophage-based product is crucial to ensure desirable and consistent therapeutic outcomes as well as therapeutic efficacy. Like chemical drugs with standard recommended protocols to test their purity, the purity test method for cell-based therapeutics is still evolving. Since the generation and scale-up process of iMACs from PSC does not involve purification steps, it is paramount to ensure iMACs homogeneity before they can be employed for therapeutic applications. In this study, we have illustrated the protocol for the scale-up of iMACs from PSC. Although the FACS analysis of macrophage-specific surface markers such as CD11b, CD14, and CD86 revealed >98% cellular homogeneity, it does not represent the cellular identity fully. Based on the single cell RNAseq data analysis of more than 6,000 cells we showed cellular homogeneity of iMACs and, have demonstrated the utility value of single-cell RNA seq for in assessing transcriptomic homogeneity of manufactured macrophages. Intrinsically, macrophages are sensitive to microenvironmental changes and respond by acquiring different functional phenotypes such as pro-inflammatory M1 macrophages to anti-inflammatory/regulatory M2 macrophages to M1-M2 intermediate phenotype (Biswas and Mantovani, 2010; Liu et al., 2020). During the differentiation and scale-up process in cultures, cells are exposed to various growth environments such as growth factors, paracrine, or autocrine soluble factors, which may induce activation of individual cells within the population of macrophages and affect the end-phenotype and function. Several studies have reported stochastic variations in the expression of genes in a similar population of cells and its impact on cell fate decisions, regenerative potential, and biological activity (Biswas and Mantovani, 2010; Das et al., 2015; Perciani and MacParland, 2019). Therefore, besides evaluating by surface markers, single-cell transcriptomic analysis may further ensure cellular purity and homogeneity.

This study used single-cell RNAseq analysis to uncover the transcriptomic homogeneity in the manufactured macrophages. Although the statistical analysis set with PC15 revealed nine dividual clusters of hESC-iMAC and seven clusters of hiPSC-iMAC, we could not distinguish the groups based on the pattern of DEGs in both the data sets. In hESC-iMAC data set, there were no significant DEGs present in clusters 0, 1, 2, and 4, which accounted for more than 75% of the population. The only distinguishable cluster with 51 DEGs contained 19 cells, which amounts to 0.6% of the total cells. Thus, the low number of DEGs in over 75% of iMACs population reflects high cellular homogeneity. Similar to hESC-iMACs, there was no significant DEGs in clusters 0, 1, 2, and 4 of hiPSC-iMAC indicating 82.7% of iMACs with similar transcriptomic profile. There was less than five DEGs in clusters 5 and 6. The cluster 3 accounts for 8.7% of the total cells and showed 11 DEGs. Based on the GO analysis of DEGs in cluster 3, we speculated that these cells are proliferating.

Recently, several clustering-based methods have been proposed to identify distinct cell populations, and their sensitivity and computational time are different across different datasets (Krzak et al., 2019). We used Seruat package and ILOReg package to identify differentially expressed genes. We found that DEGs were differently found in each method however, both data set indicated high degree of iMAC’s transcriptomic homogeneity.

We also explored which manufactured macrophage population is more similar to human blood monocyte-derived macrophages (hMDM). We found that genetic markers such as CD45, CD14, CD16, FCGR1A (CD64a), CD68, and CD71, which are commonly associated with hMDM (GEO GSE138398) were generally expressed to higher levels in hiPSC-iMACs compared to hESC-iMACs (Figures 5D,E; Supplementary Figure S3A). In addition, the gene panel of CD68, CD86, TLR2, and TLR4 associated with M1 macrophages were also elevated in hiPSC-iMAC compared to hESC-iMAC (Figures 1A, 5D,E). Our data suggest that iMAC derived from hiPSC were more comparable to primary human blood monocyte-derived macrophages. We also compared the single-cell RNA seq data of human peripheral blood mononuclear cells (PBMC) (Available at: https://support.10xgenomics.com/single-cell-gene-expression/datasets/3.0.0/pbmc_10k_protein_v3) with the RNA seq data of hiPSC-MACs and hESC-iMACs. We found that both hiPSC-iMACs and hESC-iMACs were clustered with blood myeloid cell population (Supplementary Figures S3B,C) and none of iMACs clustered with any other cell type present in PBMC (such as T, B cells). Because the peripheral blood monocytes are less differentiated cells compared to blood monocyte-derived macrophages, the PBMC monocytes and hiPSC-iMACs were distinct within the myeloid cell population.

Although with limited success, autologous macrophages have been used in clinical trials to treat solid tumors (Andreesen and Hennemann, 1991; Lee et al., 2016). We learned from these clinical studies that the donor-derived macrophages are heterogeneous and display varied functional plasticity, which could be partly attributed to differences in the intrinsic biological activity of differentiated macrophages either due to effects of age or the systemic effects of the disease itself. Recent advances in cell engineering have shown the possibility of generating a homogeneous population of cells from PSC on a large scale and allowing for allogeneic transplantation. We found that our manufacturing method consistently yielded high proportions of transcriptomically homogenous macrophage-like cells from hPSC. Our study further underscores the importance of global transcriptomic profiling by single-cell RNA-seq method for characterizing biological homogeneity of manufactured macrophages for cell based therapeutic applications. The RNA-Seq dataset of macrophages derived from hESC or hiPSC can be highly useful for comparative analysis with other cell source-derived macrophages and can aid researchers in the development of therapeutic macrophages for regenerative medicine.

## Data Availability

The datasets presented in this study can be found in online repositories. The names of the repository/repositories and accession number(s) can be found in the article/Supplementary Material.
