# Supplementary figures and images for: Single-Cell RNA Sequencing of Human Pluripotent Stem Cell-Derived Macrophages for Quality Control of The Cell Therapy Product
Source: Front Genet. 2022 Jan 31;12:658862. doi: 10.3389/fgene.2021.658862 (PMC8841343; doi:10.3389/fgene.2021.658862)

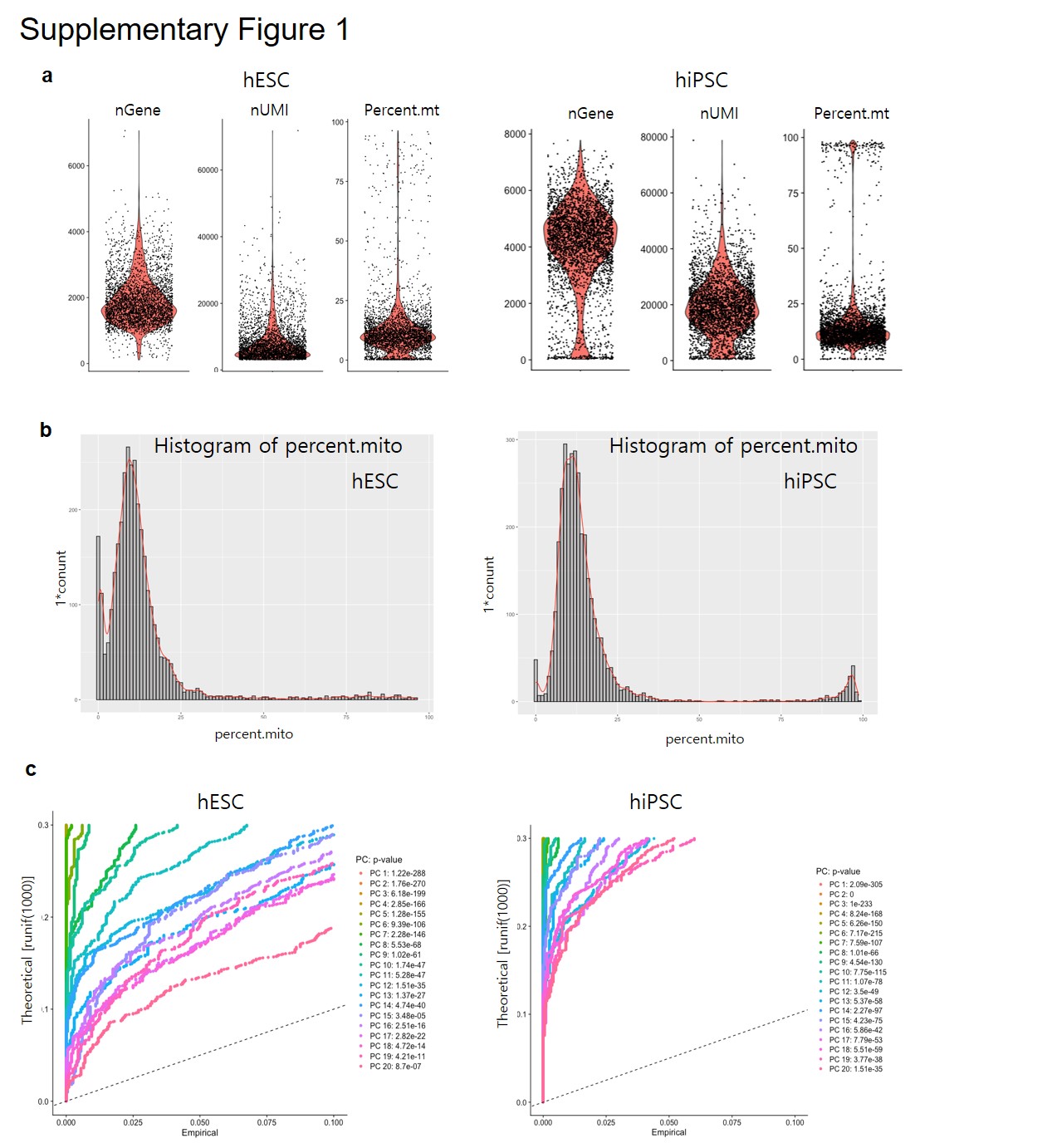

Supplement: Supplementary file 2 [file Image1.JPEG]

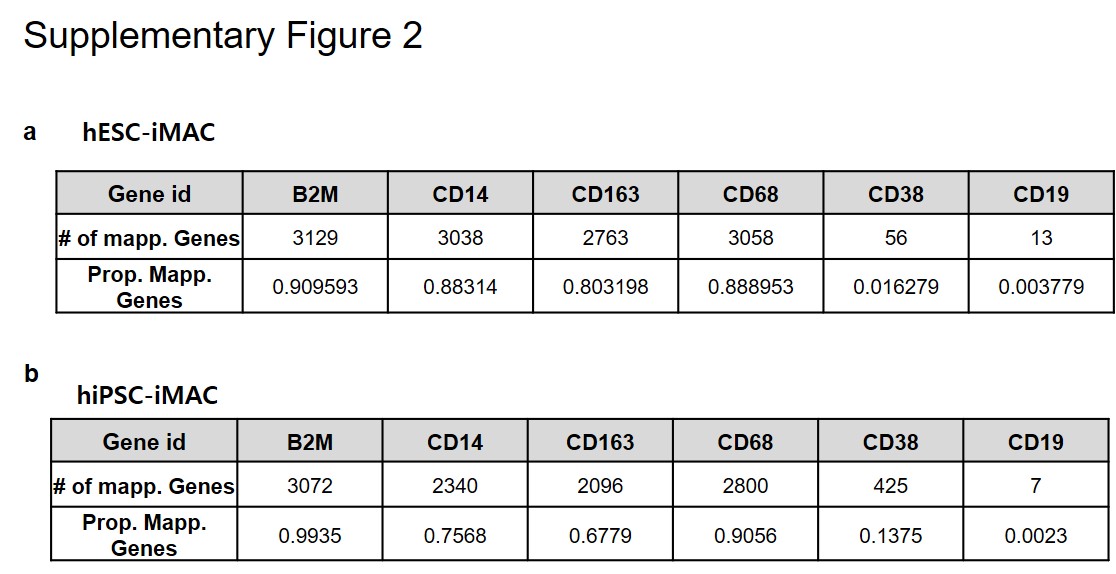

Supplement: Supplementary file 3 [file Image2.JPEG]

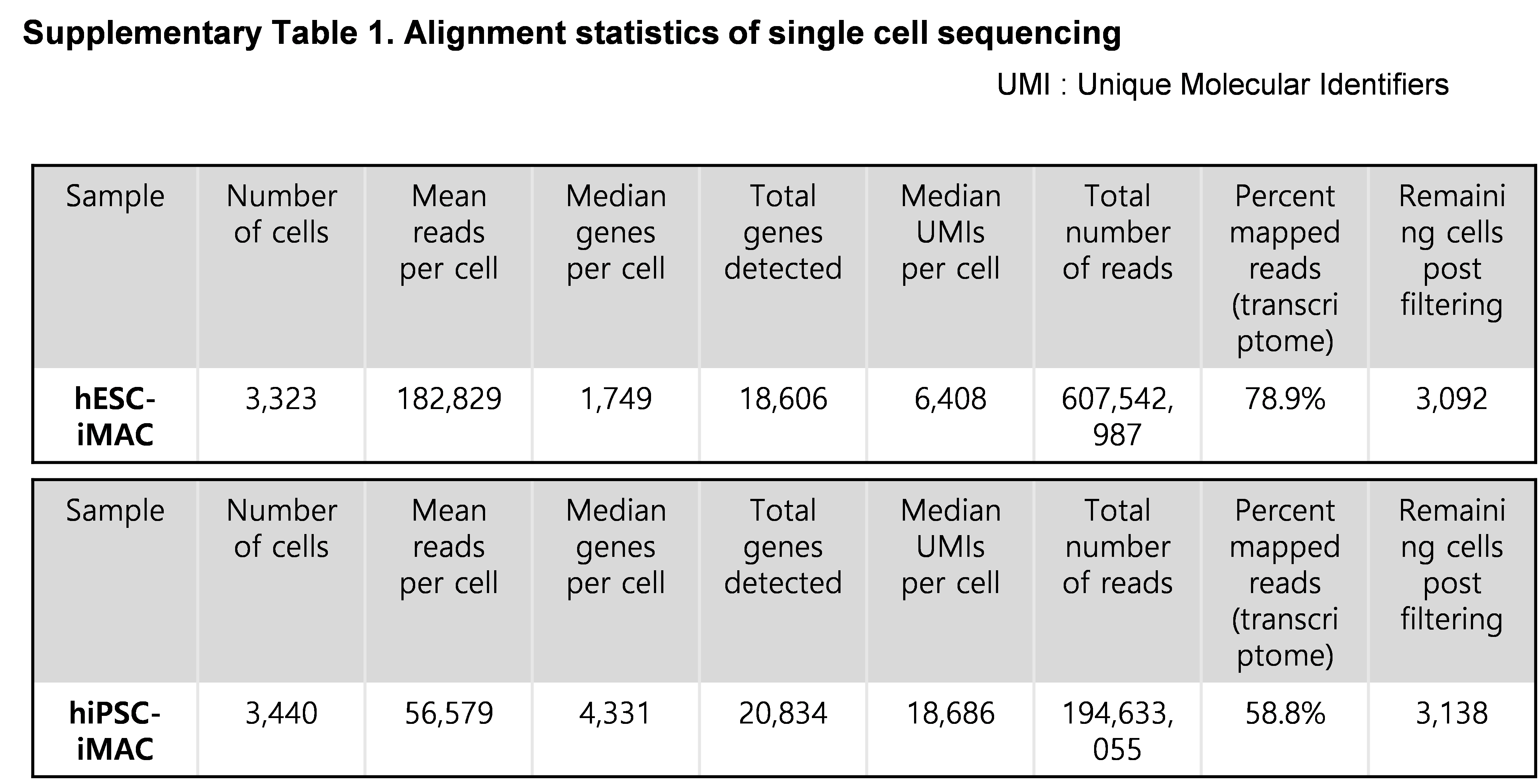

Supplement: Supplementary file 4 [file Image4.PNG]

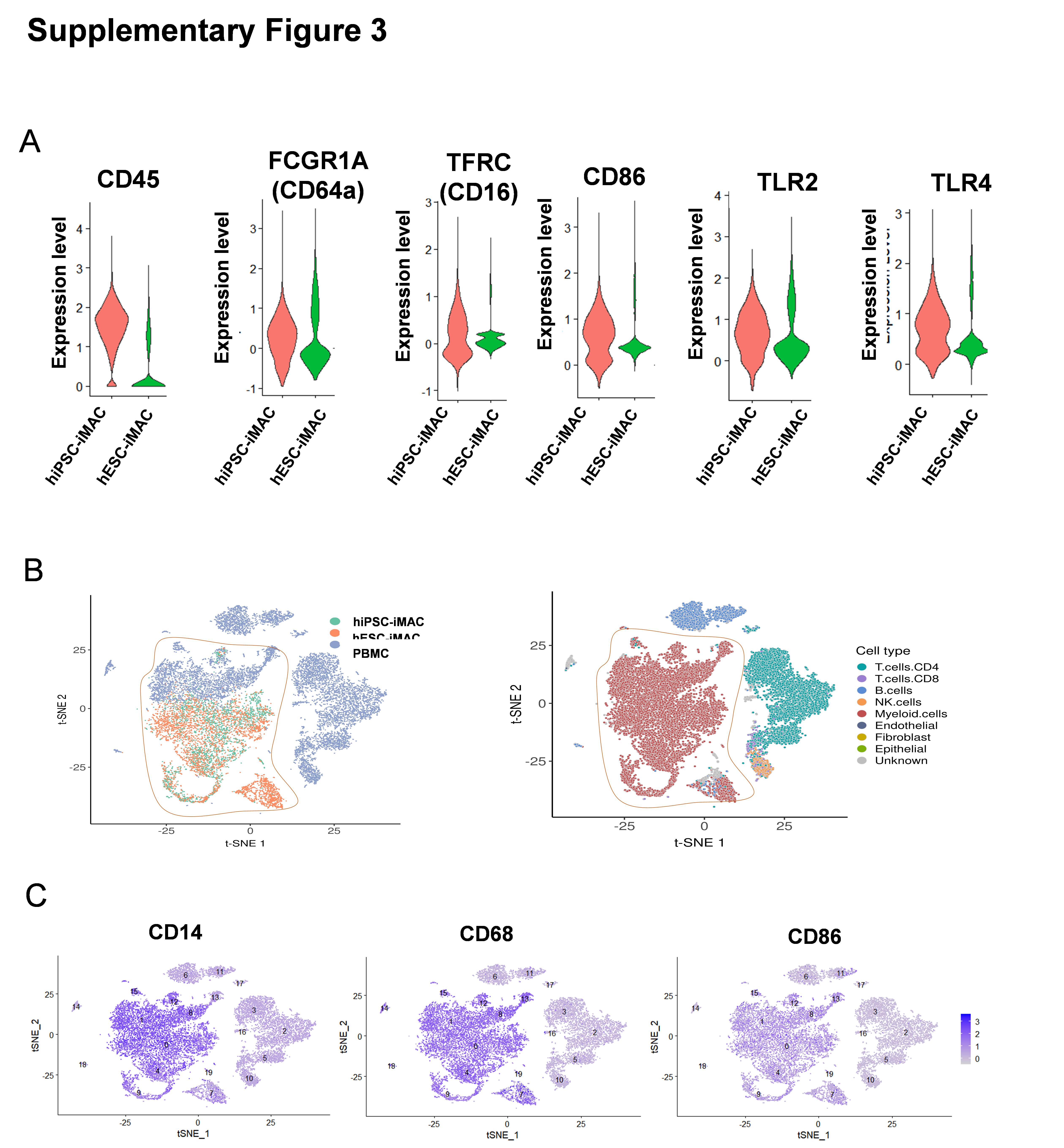

Supplement: Supplementary file 6 [file Image3.PNG]
